# Evaluation of Intradural Stimulation Efficiency and Selectivity in a Computational Model of Spinal Cord Stimulation

**DOI:** 10.1371/journal.pone.0114938

**Published:** 2014-12-23

**Authors:** Bryan Howell, Shivanand P. Lad, Warren M. Grill

**Affiliations:** 1 Duke University, Department of Biomedical Engineering, Durham, NC, United States of America; 2 Duke University, Department of Electrical and Computer Engineering, Durham, NC, United States of America; 3 Duke University, Department of Neurobiology, Durham, NC, United States of America; 4 Duke University, Department of Surgery, Durham, NC, United States of America; University of Pittsburgh School of Medicine, United States of America

## Abstract

Spinal cord stimulation (SCS) is an alternative or adjunct therapy to treat chronic pain, a prevalent and clinically challenging condition. Although SCS has substantial clinical success, the therapy is still prone to failures, including lead breakage, lead migration, and poor pain relief. The goal of this study was to develop a computational model of SCS and use the model to compare activation of neural elements during intradural and extradural electrode placement. We constructed five patient-specific models of SCS. Stimulation thresholds predicted by the model were compared to stimulation thresholds measured intraoperatively, and we used these models to quantify the efficiency and selectivity of intradural and extradural SCS. Intradural placement dramatically increased stimulation efficiency and reduced the power required to stimulate the dorsal columns by more than 90%. Intradural placement also increased selectivity, allowing activation of a greater proportion of dorsal column fibers before spread of activation to dorsal root fibers, as well as more selective activation of individual dermatomes at different lateral deviations from the midline. Further, the results suggest that current electrode designs used for extradural SCS are not optimal for intradural SCS, and a novel azimuthal tripolar design increased stimulation selectivity, even beyond that achieved with an intradural paddle array. Increased stimulation efficiency is expected to increase the battery life of implantable pulse generators, increase the recharge interval of rechargeable implantable pulse generators, and potentially reduce stimulator volume. The greater selectivity of intradural stimulation may improve the success rate of SCS by mitigating the sensitivity of pain relief to malpositioning of the electrode. The outcome of this effort is a better quantitative understanding of how intradural electrode placement can potentially increase the selectivity and efficiency of SCS, which, in turn, provides predictions that can be tested in future clinical studies assessing the potential therapeutic benefits of intradural SCS.

## Introduction

Chronic pain is a prevalent and clinically challenging condition for which there are often not adequate treatments. For example, chronic low back pain has a lifetime prevalence between 11–84% [1], is the most common cause of lost time due to disability [2], and imposes an annual economic burden of ∼$20–$120 billion [3]. Standard treatments for chronic pain, such as physical rehabilitation, pharmaceuticals (e.g., opioids), and surgery work for some individuals; but for others who do not receive satisfactory pain relief from standard treatments, alternative approaches are required.

Spinal cord stimulation (SCS) is an alternative or adjunct therapy for treating chronic pain [4], where an implanted pulse generator delivers electrical pulses to an electrode array placed in the epidural/extradural space. SCS is FDA approved for treating chronic low back and limb pain, including complex regional pains syndromes I and II and failed-back surgery syndrome [5], and it is currently being investigated for other chronic pain conditions, including ischemic limb pain, angina, and pain from peripheral neuropathy [6], [7]. SCS is based on the gate-control theory of pain [8], which postulates that activation of cutaneous (Aβ) nerve fiber collaterals in the posterior/dorsal column (DC) of the spinal cord synaptically inhibits projection neurons in the dorsal horn, which transmit pain-related information to the brain [9]. Although the gate-control theory is still an incomplete model of pain processing in the spinal cord [10]–[13], it predicts well the analgesic effects of SCS [14], [15]. Activation of Aβ fibers elicits paresthesia in the corresponding dermatome, and it is generally accepted that SCS requires the evoked paresthesia to overlap the painful area [16].

Despite substantial clinical success [5], [6], SCS is still prone to failures: the three most common being lead breakage, lead migration, and the inability to activate the target DC fibers [6], [17]. Currently, there are two electrode designs used in SCS: a percutaneous (PERC) design, consisting of 4–8 annular electrodes distributed along a flexible cylindrical shaft, which is inserted with a hypodermic needle; and a laminectomy/paddle design (LAM) implanted during a surgical laminectomy, consisting of a grid of 4–20 rectangular electrodes on a flexible planar substrate. PERC designs are less invasive but more prone to lead migration than LAM designs [17], while LAM designs, although more invasive and prone to fracture [17], are, in practice, more energy efficient and evoke fewer side effects than PERC designs [18]–[20].

Activation of DC fibers in the dermatomes associated with pain is often limited by the onset of discomfort. Since discomfort is associated with activation of nearby Aβ fibers in the dorsal roots (DR) [21], [22], successful SCS is thought to depend on activation of DC fibers without activation of DR fibers, which we term selectivity. Selectivity can be improved using longitudinal (rostral-caudal) and transverse (medial-lateral) bipolar and tripolar electrode configurations [23]–[26], mitigating the sensitivity to malposition or migration of the electrode array. However, there is a limit to selectivity with extradural placement due to the large distance between the electrodes and the neural elements, and the high conductivity of the intervening cerebrospinal fluid.

The shunting effects of the CSF can be surmounted by placing the electrodes below the dura mater and adjacent to the dorsal aspect of the spinal cord [27], [28]. Directing less current through the CSF and more through the spinal cord results in a potential distribution with isopotential contours more closely spaced on the side of the electrode facing the spinal cord. As a result, there is a greater difference in the magnitude of the potentials between the DC and DR fiber populations. DR fibers, however, are more excitable than DC fibers of the same diameter because of their curved geometry and entry into the white matter from the CSF, where the tissue conductivity changes drastically [22]. Therefore, if increasing the proximity of the electrode to the spinal cord does not substantially increase the excitability of DR fibers over DC fibers, subdural/intradural SCS may have substantially greater selectivity than extradural SCS.

Intradural electrode placement was used when SCS devices were first implanted in humans. Because intradural electrodes are more prone to movement than extradural electrodes, and because the first electrodes had a limited number of contacts, adjustments following implantation were difficult [29]. Moreover, multi-level laminectomies and large dural openings introduced additional risks, including exposure to general anesthesia, cerebral spinal fluid (CSF) leaks, and increased postoperative pain and tissue scarring [30]. For these reasons, intradural SCS was abandoned. However, with the advent of new techniques [31] and electrode designs [27] for addressing movement of the spinal cord and electrode, the possibility of intradural SCS is being revisited.

The goal of this study was to develop a computational model of SCS. We compared the stimulation thresholds predicted by the model to stimulation thresholds measured intra-operatively and used the model to compare the performances of extradural and intradural SCS. The outcome is a quantitative understanding of how intradural electrode placement can potentially increase the selectivity and efficiency of SCS, which, in turn, provides predictions that can be tested in future clinical studies assessing the potential therapeutic benefits of intradural SCS.

## Methods

### Volume conductor model of the spinal cord and SCS electrode

**Figure 1:**
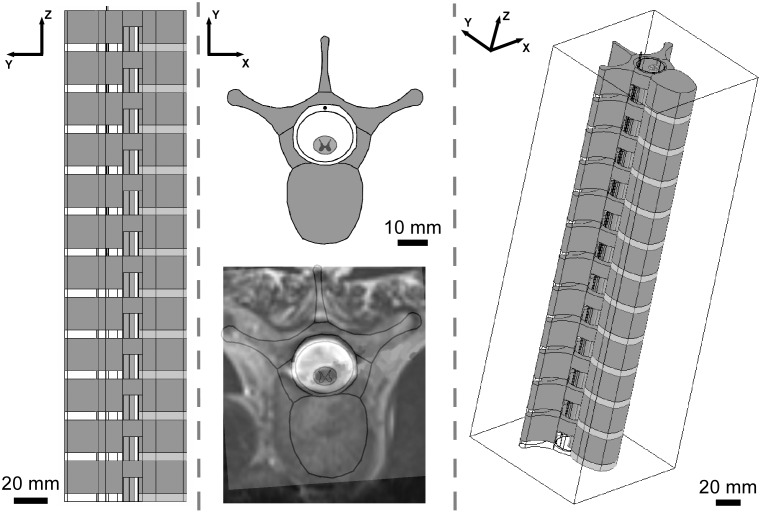
Finite element model of the spine and spinal cord. Sagittal (*left*), transverse (*middle-top*), and 3D (*right*) views of the modeled spine, which consists of 12 vertebrae spaced by disks and a dural sac and spinal cord that traversed the spinal canal. The transverse section of the modeled spine is overlaid with a transverse magnetic resonance image of the spine of Patient 2 (*middle-bottom*).

The finite element method (FEM) was used to construct a volume conductor model of the spinal cord and an implanted three-contact percutaneous electrode array in COMSOL Multiphysics v3.4 (COMSOL Inc., Burlington, MA) (Fig. 1). Three electrodes were chosen to enable monopolar (−), bipolar (+ −), and tripolar (+ − +) configurations. The model spine consisted of 12 vertebrae, thoracic level 3 (T3) to lumbar level 2 (L2), spaced by disks, and a dural sac and spinal cord that traversed the spinal canal. The model spine was placed within a rectangular prism of homogeneous soft tissue that was large enough (100 mm×100 mm×300 mm) to behave as an infinite conducting medium (Fig. 1). Spinal dimensions were consistent with those of an adult human lower thoracic/upper lumbar spine [32]–[35]. All tissues were modeled as purely resistive [36], [37], with electrical conductivities from published data (Table 1).

**Table 1:** Electrical conductivity of tissues represented in the volume conductor model of spinal cord stimulation.

Tissue	Conductivity (S/m)	Reference
White matter, longitudinal	0.60	[37]
White matter, transverse	0.083	[37]
Grey matter	0.23	[38]
Cerebrospinal fluid	1.8	[39]
Dura mater	0.030	[40]
Extradural space^a^	0.20	[41]
Vertebral bone	0.02	[42]
Intervertebral disc	0.65	[43]
Muscle^b^	0.20	[44]

a The lumped conductivity of the extradural space was assumed to be similar to that of muscle.

b Tissue surrounding spine.

A Delaunay triangulation algorithm was used to discretize the FEM model into a graded mesh of Lagrange tetrahedral cubic elements, where the mesh density was greatest near the electrode surfaces, spinal cord, and dura mater. The shaft of the array was assumed to be perfectly insulating and was modeled as a boundary layer with zero normal current density (*i.e.,* Neumann boundary condition). Monopolar stimulation was modeled by applying potentials of 1 V and 0 V (*i.e.,* Dirichlet boundary conditions) on the surface of the stimulation electrode and outer surface of the tissue volume, respectively. Bipolar and tripolar configurations were modeled by applying potentials of +1 V and −1 V and +1 V, −1 V, and +1 V to either 2 or 3 consecutive contacts, respectively. In the bipolar and tripolar cases, the cathode served as the return, and the outer tissue boundary was assumed to be perfectly insulating. The electric potentials (Φ) generated in the tissue by the electrode array were calculated by solving Laplace’s equation (Equation 1):
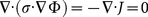
(1)where *σ* is the conductivity tensor, and *J* is the current density. The applied current was calculated by integrating the current density (Equation 2) on the surface of electrodes with positive applied potentials (*i.e.,* anodes):
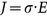
(2)where, *E*is the electric field. The lumped resistance of the tissue, also known as the series or access resistance (*R_a_*), was calculated by dividing the voltage between the anode and cathode by the applied current.

### Population model of dorsal column and dorsal root axons

We used the NEURON (v7.1) simulation environment to implement cable models of dorsal column (DC) and dorsal root (DR) fibers, and to calculate their response to modeled SCS. Two different non-linear cable models of axons with Hodgkin-Huxley-type ion channels were used: a mammalian axon model with perfectly insulating myelin [45], the SW model, which was used in previous computational models of SCS [20]–[26], [40], [46]–[48]; and a more detailed model of a myelinated mammalian axon, the MRG model [49], which takes into account the structure and electrical properties of the myelin.

Because the myelin in the SW model is assumed to be perfectly insulating, only the nodes of Ranvier (NoR) and internodal resistance were modeled. The membrane at each NoR contained a parallel combination of a nonlinear sodium conductance, a linear leakage conductance, and a membrane capacitance [45]. In the MRG model, each NoR contained fast and persistent sodium conductances, a slow potassium conductance, a linear leakage conductance, and a membrane capacitance; and the membrane underneath the myelin contained a linear leakage conductance and a membrane capacitance [49].

**Figure 2:**
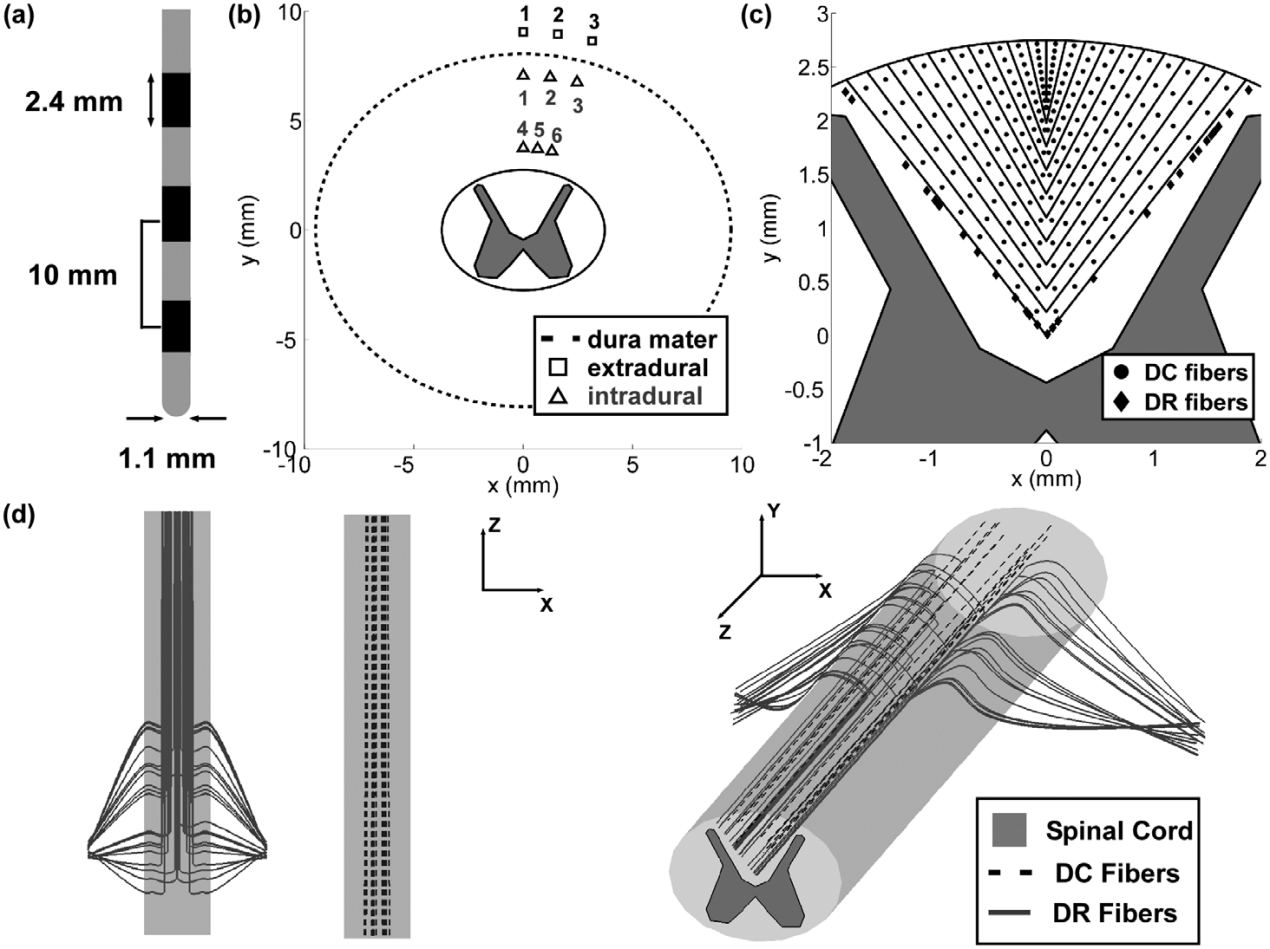
Placement of model dorsal column (DC) fibers, model dorsal root (DR) fibers, and the stimulation electrode. (a) The modeled percutaneous array was placed in (b) nine different locations: three extradural locations and six intradural locations. (c) Transverse view of the spinal cord showing the locations of the modeled DC fibers and DR fibers within the dorsomedial white matter. (d) Planar dorsal and lateral views (*left*) and 3D view (*right*) illustrating modeled DC fibers and DR fibers.

The dorsomedial white matter of the spinal cord (*i.e.,* between the dorsal boundary of the spinal cord and the grey matter) was split into 11 dermatomes based on the mediolateral segmental lamination of DC fibers [50], [51]. A total of 200 DC fibers were bilaterally distributed–10 fibers within each of the 10 most medial dermatomes on either side of the transverse midline (Fig. 2c) – representing collaterals that originated from distal, caudal DR fibers, which were not modeled. An additional 200 DC fibers were bilaterally distributed in the lateral-most dermatome, and these fibers were attached to the proximal end of 200 corresponding DR fibers. DR fibers descended from the dorsal aspect of the spinal cord in a ventrolateral direction (*i.e.,* via the rootlets) and exited the spine through the intervertebral foramina [22] (Fig. 2d).

The diameters of myelinated fibers in the dorsomedial white matter range between 1–15 µm [52]. Although the vast majority (>60%) of the fibers have diameters between 1–7 µm [53], prior computational modeling studies have shown that fibers as large as 12 µm are activated within the therapeutic range of in SCS [21]. We considered nine separate populations of 200 DC fibers with diameters of 3, 6, 9, 12 and 15 µm; and nine separate populations of 200 DR fibers with the same diameters.

Potentials were calculated using the maximum possible number of cubic elements (∼1.34 million) with 8 GB of memory. Refinement of the FEM mesh (*i.e.,* splitting elements into smaller elements) from ∼635,000 to 1.34 million cubic elements resulted in errors of <1% in the potentials and corresponding stimulation thresholds of the modeled fibers, where error was defined as the mean absolute relative difference between the refined solution and the solution prior to refinement. Similarly, doubling the tissue volume yielded errors of <2% and <1% in the potentials and stimulation thresholds of the modeled fibers, respectively.

### Clinical protocol

Five subjects were enrolled in a controlled exploratory study of acute SCS trial system implantation (NCT02020460 on clinicaltrials.gov). The Duke University Institutional Review Board approved the study, and all participants gave written informed consent.

Subjects were injected with a local anesthetic in the lower back, and a sedative was administered intravenously. Fluoroscopy was used to guide placement of an eight-contact extradural trial lead, the Spencer Probe Depth Electrode (AD-TECH Inc., Racine, WI) (Fig. 2a), in the extradural space and pick five consecutive contacts that spanned the vertebral levels of interest, T8-T10. The extradural array was connected to an external stimulator (MTS, St. Jude Medical, Saint Paul, MN), and stimulation was delivered in a bipolar configuration between the two contacts closest to T8, where the rostral cathode and caudal anode were proximal and distal to T8, respectively. Stimulation waveforms were current-regulated, asymmetric pulses with a short 300 µs positive phase followed by a long 700 µs negative phase delivered at 60 Hz.

A staircase paradigm was used to determine sensory and discomfort thresholds. First, a reference for paresthesia was established: the stimulation amplitude (I_A_) was increased in 1 mA increments from 0 mA until the subject reported a sensation (i.e., S), and I_A_ was decreased in decrements of 1 mA until S was no longer perceived. The subject was asked to describe orally the location of S. Second, I_A_ was increased in 0.1 mA increments until S was reported, defined as I_S1_. Third, I_A_ was decreased in 0.1 decrements until S went away, defined as I_S2_. Fourth, I_A_ was increased in 0.1 mA increments until S was reported, defined as I_S3_. The sensory threshold was defined as the average of I_S1_, I_S2_, and I_S3_. Finally, I_A_ was increased in increments of 0.1 mA until the subject reported discomfort and/or pain, which was defined as the discomfort threshold. As I_A_ was increased, the subject was asked to describe orally any additional locations of S.

After sensory and discomfort thresholds were measured in the extradural case, fluoroscopy was used to place the AD-TECH array within the dura in a fashion similar to insertion of a standard lumber drain catheter, and the array was connected to the external stimulator. As in the extradural case, current-regulation stimulation was delivered at 60 Hz between the two contacts most proximal to T8, and the staircase paradigm was used to determine sensory and discomfort thresholds.

### Model evaluation

We constructed five models of SCS based on the individual spinal cord dimensions of patients that had undergone acute intraoperative evaluation of extradural and intradural SCS. Preoperative magnetic resonance imaging (MRI) scans of the corresponding patients were used to measure the geometries of the spinal cord and dural sac, and the position of the cord within the sac (Table 2). The geometry of the modeled spinal column did not vary across patients and reflected the geometry of an average adult human lower thoracic/upper lumbar spine [32]–[35] (Fig. 1).

**Table 2:** Spinal cord geometry from individual patients^a^.

Patient	Spinal CordDimensions (mm)		CSF SpaceDimensions (mm)		Spinal CordPlacement^b^
	Mediolateral	Ventrodorsal	Mediolateral	Ventrodorsal	
1	7	6	19	16	center^c^
2	8	7	21	18	ventral^d^
3	8	6	20	14	center
4	9	6	18	13	center
5	8	6	18	15	ventral

a Geometries were measured from MRI images (at 1.5 Tesla) with a resolution of 1 mm.

b Placement with respect to midline.

c Spinal cord center placed at the center of the elliptical shell defining the dura mater.

d The spinal cord center was twice as far from the dorsal interior surface of the dura mater as it was from the ventral interior surface of the dura mater.

Patient-specific models were evaluated at nine different electrode locations (Fig. 2b): three extradural locations 1 mm above the dura, three intradural locations 1 mm below the dura, and three intradural locations 1 mm above the spinal cord, where points one, two, and three in each set had lateral (clockwise) offsets of 0°, −10°, and −20° from the transverse midline, respectively.

Clinical conditions were emulated by stimulating the model DC and DR fibers with a 300 µs monophasic rectangular pulse, consistent with typical pulse widths (175–600 µs) used in SCS [54]. The cathode was proximal to T8, and the distal anode was rostral to the cathode. Due to linearity of the solution, the potentials at a given stimulus amplitude were calculated by multiplying the base (monopolar, bipolar, or tripolar) solution by a scalar. The stimulation threshold voltage for each fiber was calculated using a bisection algorithm (relative error <1%). The stimulation threshold current was calculated by dividing the threshold voltage by *R_a_*, and input-output curves of the number of activated model nerve fibers as a function of the stimulation amplitude and stimulation power were constructed.

The stimulation thresholds of the MRG and SW model fibers were compared to the clinically measured sensory thresholds, when the patient first reported a paresthesia, and the discomfort threshold, when the patient first reported pain or discomfort. We used the following procedure to determine the percentage and diameter of DC fibers that most closely matched the clinical findings. First, for each diameter, we calculated the percentage of DC fibers activated at the sensory and discomfort thresholds. Next, because two hundred model DC fibers were evenly split across 20 laminae (Fig. 2c), the clinical findings were translated into an expected percent activation by assuming that paresthesia in one dermatome on one side of the body corresponded to activation of 5% of the modeled DC fibers. The DC fiber population (of a given diameter) that best matched clinical findings was defined as the one whose percent activation at the clinical thresholds most closely matched the expected percent activation based on the number of dermatomes reported at the clinical thresholds.

The model of SCS was used to quantify the efficiency and selectivity of stimulation. Stimulation efficiency was quantified by calculating the average electrical power (Equation 3) consumed during the stimulation pulse to activate the target DC fibers:
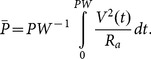
(3)where *V(t)* is the applied voltage, and *PW* is the duration of the rectangular pulse.

Selectivity was quantified using two different metrics: neural-element selectivity was analyzed by calculating the percent of DC fibers activated with no activation of DR fibers (DC_0_) or with activation of a certain percentage (X) of DR fibers (DC_X_), and dermatome selectivity was analyzed by determining if DC fibers in target dermatomes could be activated without activation of DC fibers in non-target dermatomes. Since the computational models were based on data from patients receiving SCS for the treatment of chronic low back pain, we chose the low back dermatomes, L2–L5, to assess dermatome selectivity.

### The effect of electrode geometry

**Figure 3:**
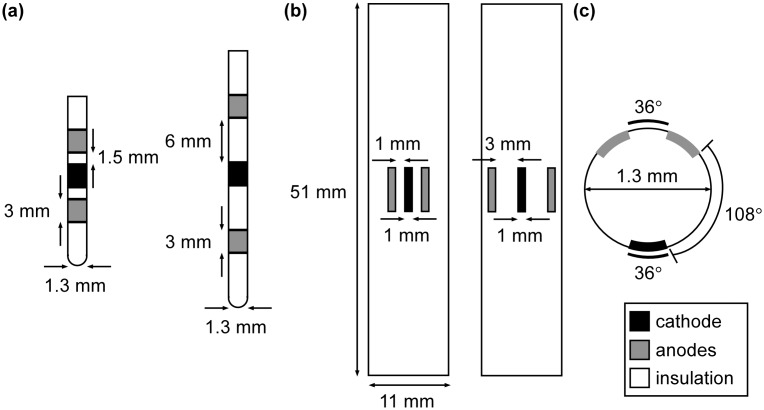
Five SCS electrode designs evaluated with the computational model. (a) Medtronic Models 3776/3876 (*left*) and 3777/3877 (*right*) in longitudinal tripolar configurations. (b) St. Jude Medical Penta in two transverse tripolar configurations. (c) A transverse view of a novel percutaneous lead with an azimuthal array of electrodes in a tripolar configuration. Inactive contacts were not represented.

In addition to analysis with the lead (AD-TECH Spencer Probe Depth Electrode) used in the clinical experiments, simulations were conducted to test the extradural and intradural performance of five different electrode designs (Fig. 3): LT-1.5 and LT-6, which are two percutaneous leads, Medtronic Models 3776/3876 and 3777/3877 (Medtronic Inc., Minneapolis, MN), in longitudinal tripolar configurations with an inter-electrode spacing (IES) of 1.5 mm and 6 mm, respectively; TT-1 and TT-3, which are the St. Jude Medical Penta (St. Jude Medical, Saint Paul, MN) in two transverse tripolar configurations with an IES of 1 mm and 3 mm, respectively; and our own novel design.

TTs are believed to have greater selectivity than LTs because their distal anodes hyperpolarize the DR fibers, termed anodal shielding [20], [25], [26]. However, we believed that the superior selectivity of TTs over LTs arose from their ability to steer current away from DR fibers so that the potentials decayed more rapidly with increasing distance from the cathode. To test this, we modeled a fifth tripolar design, a percutaneous azimuthal array in an angular tripolar (AT) configuration, which had a cathode adjacent to the dorsomedial aspect of the spinal cord and anodes that directed current away from the cord, toward the dorsal aspect of the dura (Fig. 3). The performance of all five designs was assessed with the each electrode array placed along the transverse midline, extradurally 1 mm above the dura mater and intradurally 1 mm above spinal cord (Fig. 2b).

### Statistical analyses

The computational models of Patients 1–5 account for the effects of anatomical variability in the spinal geometry across patients. Yet, five sets of spinal geometries were not sufficient to determine if the geometrical parameters and subsequent output metrics (e.g., R_a_ and DC_0_) varied normally across patients, so we used a Kolmogorov-Smirnov (K–S) test to determine differences in the distributions of the output metrics across Patients 1–5. The K–S test assumes nothing about how the output metrics are distributed, and all K–S tests were run at a significance level of 5% (α = 0.05).

Linear regression and subsequent calculation of coefficients of determination (r^2^) were used to determine whether the variability in the sensory and discomfort thresholds could be explained by differences in anatomical measurements across patients.

## Results

We constructed five patient-specific models of SCS, compared the model stimulation thresholds to clinical stimulation thresholds, and used the models used to quantify the efficiency and selectivity of both intradural and extradural SCS.

### Clinical findings

**Figure 4:**
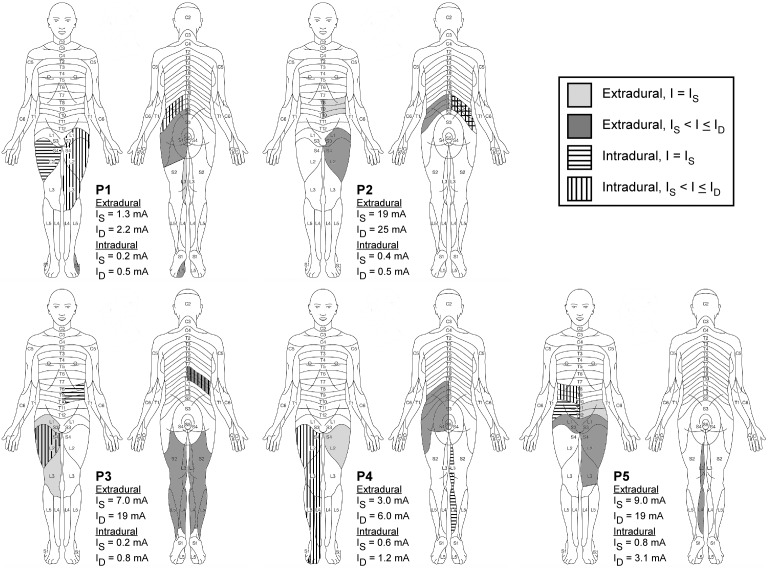
Reported locations of paresthesias in Patients 1–5. Paresthesias in each patient (P) were charted based on their oral descriptions at the sensory threshold (I_S_) and increasing amplitudes until the discomfort threshold (I_D_) was reached. Dermatome maps were adapted from a free resource on http://www.change-pain.co.uk/.

Paresthesias at the sensory thresholds of Patients 1–5 were reported in the legs, middle and lower back, buttocks, belly, and waistline region (Fig. 4). In Patients 1, 4, and 5, the location of paresthesia differed between the intradural and extradural cases; whereas in Patients 2 and 3, the locations of the paresthesia in the two cases overlapped. In all patients, as the stimulation amplitude increased, paresthesias were reported in additional locations. The quality of the paresthesias and the location of discomfort were not determined.

The sensory thresholds across all patients ranged from 0.2–0.8 mA and 1.3–19 mA in the intradural and extradural cases, respectively, whereas the discomfort thresholds ranged from 0.5–3.1 mA and 2.2–25 mA (Fig. 4). Distributions of the sensory and discomfort thresholds across patients were not significantly different within the intradural and extradural cases. However, between the intradural and extradural cases, the distributions of sensory (p<0.01) and discomfort (p<0.05) thresholds across patients were significantly different.

The extradural sensory thresholds showed a strong linear correlation (r^2^>0.80, p<0.07) with the ventrodorsal width of the CSF space and the ventral displacement of the spinal cord from the center of the CSF space. The intradural sensory (r^2^<0.42, p>0.18) and discomfort (r^2^<0.43, p>0.19) thresholds and the extradural discomfort thresholds (r^2^<0.56, p>0.14) showed much weaker correlations with differences in the patient geometries.

### Comparing model predictions with clinical data

**Figure 5:**
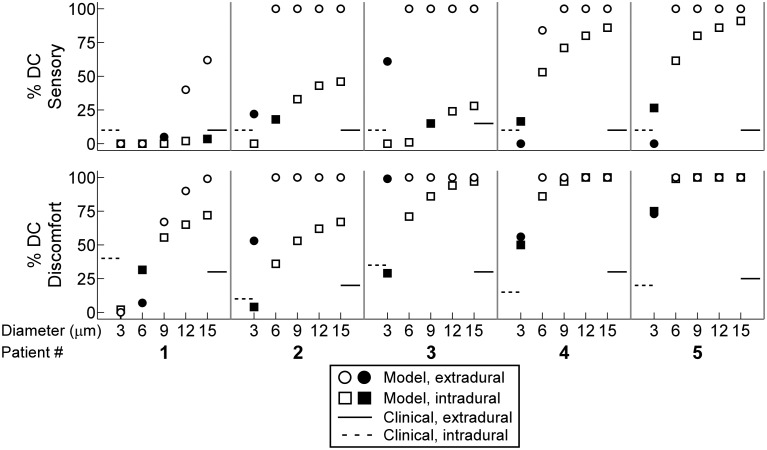
Predicting the diameter of dorsal column (DC) fibers activated based on clinical thresholds. The percentage of MRG model DC fibers (*denoted by circles and squares*) activated at the sensory (*top*) and discomfort (*bottom*) thresholds compared to the percent activation expected (*denoted by solid and dashed lines*) based on the number of dermatomes reported at the corresponding clinical thresholds (see Methods). The filled shapes indicate the fiber diameters that yielded the smallest percent difference between the former and latter cases.

We used the number of dermatomes reported by patients at the sensory and discomfort thresholds to determine the diameter of the MRG model of DC fibers that most closely matched the clinical findings. The diameter of the model DC fiber population that most closely matched the sensory thresholds differed across patients and between the intradural and extradural cases in some of the patients, and the same was true for the diameters that most closely matched the discomfort thresholds (Fig. 5). Patients 2 and 3 reported paresthesia in T8 at their sensory thresholds, but because this was not consistent across patients in the intradural and extradural cases, we did not compare the percentage and diameter of DR fibers to the sensory thresholds.

At the discomfort threshold in Patients 1–5, the locations of the paresthesias ranged from T8-S5 (Fig. 4), so we determined whether an increase in the activation of DC fibers or activation of DR fibers could predict discomfort. For all diameters except 3 µm (p = 0.03), the distributions of the percentage of DC fiber activated at the discomfort and sensory thresholds across patients were not significantly different, and for all fiber diameters, there was no significant difference between the distributions of the percentage of DC and DR fibers activated at the discomfort thresholds.

**Figure 6:**
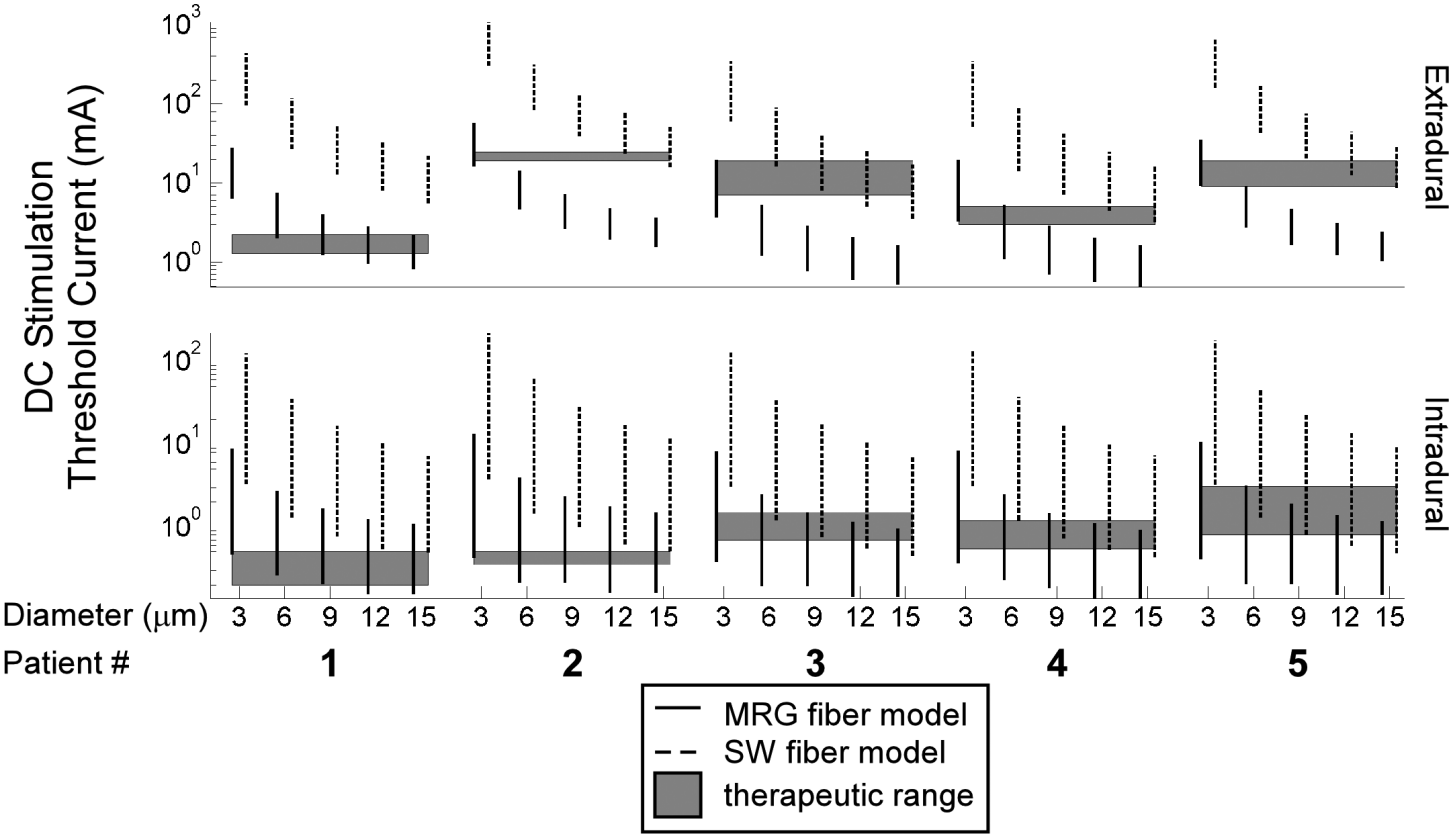
Comparing model predictions of stimulation thresholds between MRG and SW models of dorsal column (DC) fibers. Plotted are distributions of the stimulation threshold currents of DC fibers when the AD-TECH was placed 1 mm above the spinal cord and 1 mm above the dura in the intradural and extradural cases, respectively. The therapeutic range is defined as the stimulation amplitudes between the measured sensory and discomfort thresholds.

In addition to the above, we also compared the stimulation thresholds of SW models of DC fibers to the clinical stimulation thresholds. With the SW model, the diameter of the DC fiber population that most closely matched the sensory threshold was different across patients and between the intradural and extradural cases in some of the patients. In general, the stimulation thresholds predicted by the SW model were greater than the stimulation thresholds predicted by the MRG model, so compared to the MRG model, the SW model predicted that DC fibers with larger diameters were activated (Fig. 6).

Because fibers with diameters from 3–15 µm could be activated within the range of the two clinical thresholds, we used the intermediate fiber diameter, 9 µm, to quantify the efficiency and selectivity of SCS. We used the MRG model in the remainder of our analyses.

### Power efficiency of intradural SCS

For electrodes positioned along the midline, the calculated access resistance (*R_a_*) of the AD-TECH array was 1,150 Ω (mean, n = 5) 1 mm above the dura mater, 165 Ω 1 mm below the dura mater, and 153 Ω 1 mm above the spinal cord. At a given dorsal-ventral position, distributions of *R_a_* across models of individual patients for electrodes at the three lateral deviations from the midline (0°, −10°, and −20°) were not significantly different from each other, but at a given lateral deviation, distributions of *R_a_* across models of individual patients were significantly different (p<0.05) between the dorsal-ventral positions. Therefore, *R_a_* was sensitive to dorsal-ventral position, primarily between the intradural and extradural locations, and insensitive to lateral deviations.

**Figure 7:**
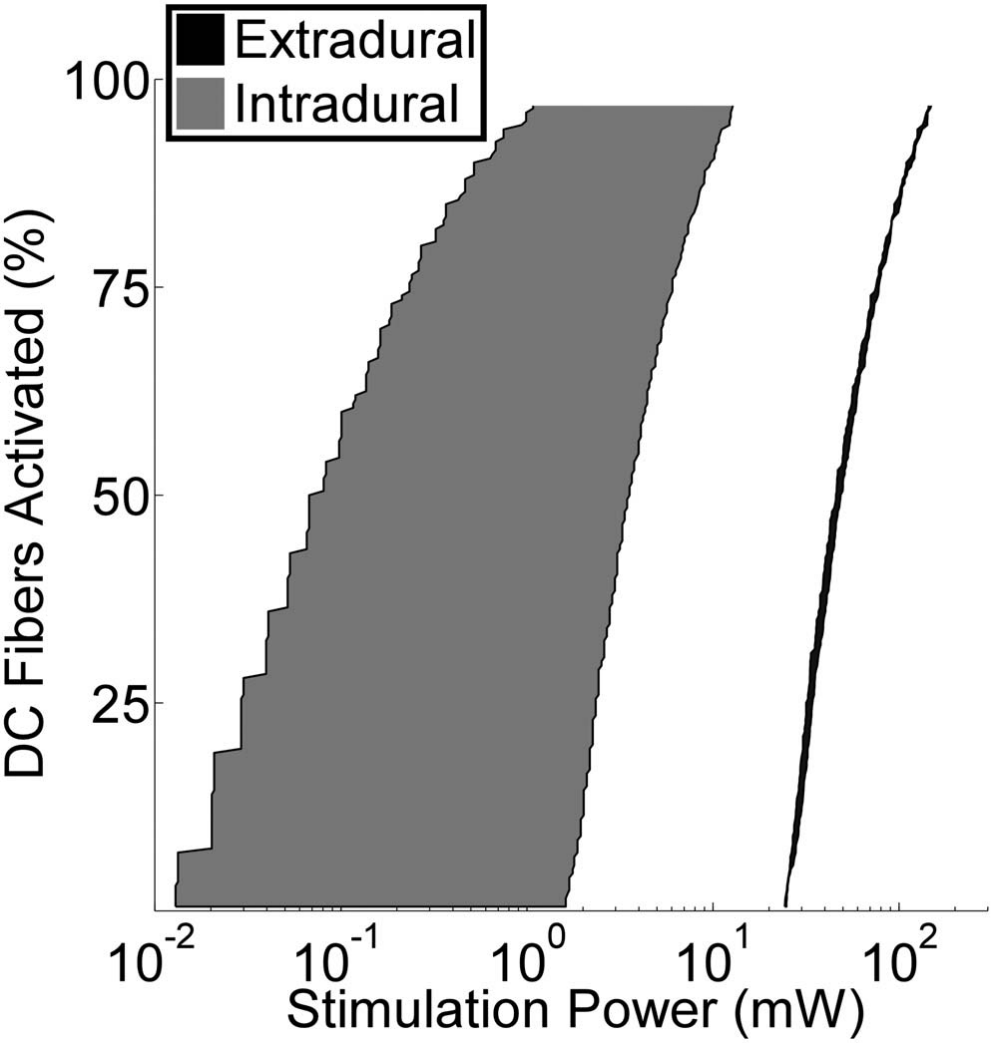
Power efficiency of extradural SCS versus intradural SCS. Average power required to stimulate the dorsal column (DC) fibers in the SCS model of Patient 2. The shaded black and grey areas encompass the range of stimulation powers calculated over the three extradural electrode locations and six intradural electrode locations, respectively.

The reduction of *R_a_* by >85% when the electrode was moved from the extradural space to the intradural space, along with the increased proximity of the electrode to the spinal cord, dramatically reduced the power required to activate DC fibers (Fig. 7). The average power required to activate half of the DC fibers at all six intradural locations was reduced >90% compared to epidural SCS at all three electrode locations.

### The selectivity of intradural SCS

**Figure 8:**
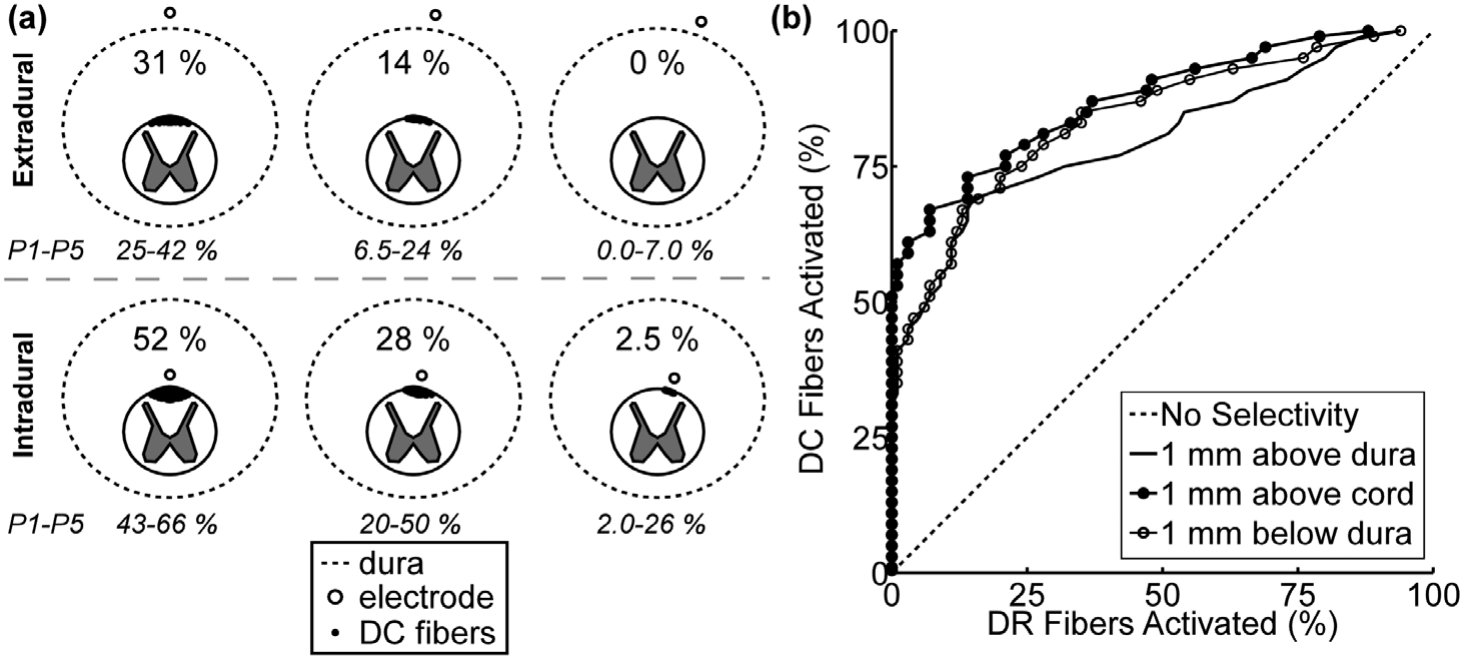
The selectivity of extradural SCS versus intradural SCS in model of Patient 5. The maximum percentage of dorsal column (DC) fibers activated with no activation of dorsal root (DR) fibers (DC_0_) when the array was placed in the extradural (*top row*) and intradural (*bottom row*) spaces at lateral deviations of 0° (*left column*), −10° (*middle column*), and −20° (*right column*). For comparison, the range of DC_0_ across all patients is shown below each panel. (b) Curves of the proportion of DC fibers activated versus proportion of DR fibers activated for three different electrode locations along the midline.

The ability to activate selectively DC fibers over DR fibers was sensitive to electrode placement. In both the extradural and intradural cases, distributions of neural-element selectivity (*i.e.,* DC_0_) across models of individual patients at 0°, −10°, and −20° from the midline were significantly different (p<0.05) from each other. DC_0_ was greatest when the lead was positioned along the midline and declined with increasing displacement of the electrode from the midline (Fig. 8a). At each of the lateral displacements from the midline, the distribution of DC_0_ across models of individual patients was significantly greater (p<0.05) with intradural placement than extradural placement. Thus, DC_0_ was greatest when the intradural electrode was located along the midline and closest to the spinal cord.

It is not known how many DR fibers must be activated to evoke discomfort in SCS and whether this number varies from patient to patient. To account for this uncertainty in what constitutes discomfort, we constructed curves of the proportion of DR fibers activated versus proportions of the DC fibers activated (*i.e.,* DC_X_). DC_X_ was greater with intradural placement than with extradural placement when the electrode was located along the midline and closest to the spinal cord (Fig. 8b).

**Figure 9:**
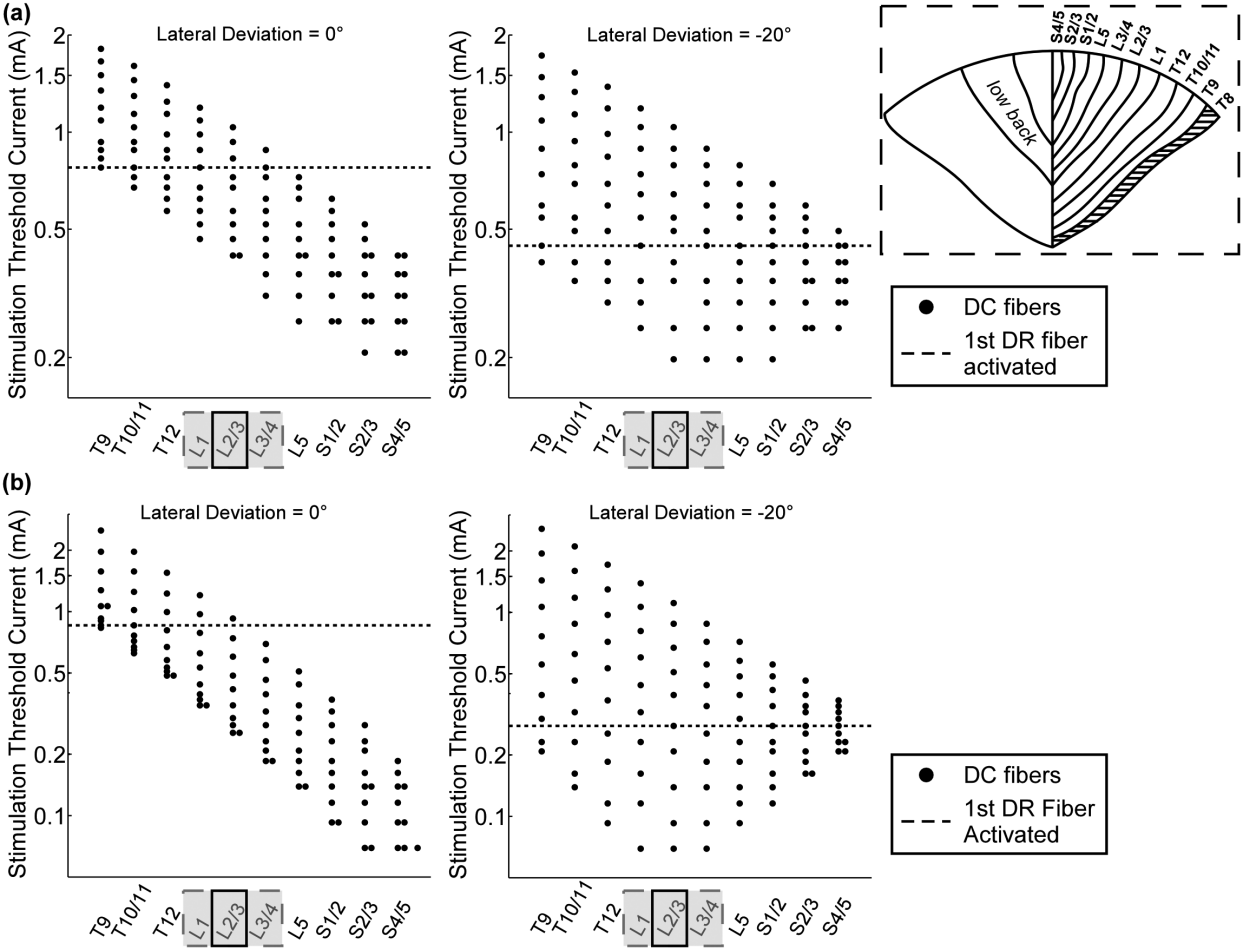
Selective activation of dorsal column (DC) fibers in the low back (L2–L5) dermatomes of Patient 1. (a) Stimulation threshold current of each of the model DC fibers, split by dermatome (*see inset*), when the AD-TECH array was placed in the intradural space and laterally displaced 0° (*left*) and −20° (*right*) from the midline. The shaded area in the inset illustrates where the Aβ collaterals of the DR fibers were located with respect to DC dermatomes at T8. (b) The same as (a), except for the angular tripole electrode geometry. The locations of the paresthesias at the sensory and discomfort thresholds are denoted by the open black rectangles and filled grey rectangles, respectively.

Activation of DC fibers in lateral dermatomes at lower amplitudes than required for activation of DC fibers in medial dermatomes was possible by displacing the electrode laterally from the midline. For example, in Patient 1, when the array was positioned along the midline, 1 mm above the cord, DC fibers in L2–L5 could not be activated without first activating DC fibers in S2–S5 (Fig. 9a). However, when the array was displaced −20° from the midline, 1 mm above the cord, DC fibers in L2–L5 could be activated but not without co-activation of DC fibers in S1 and S2 (Fig. 9a). Similar results were obtained in Patients 2–5.

### The effect of electrode geometry

**Figure 10:**
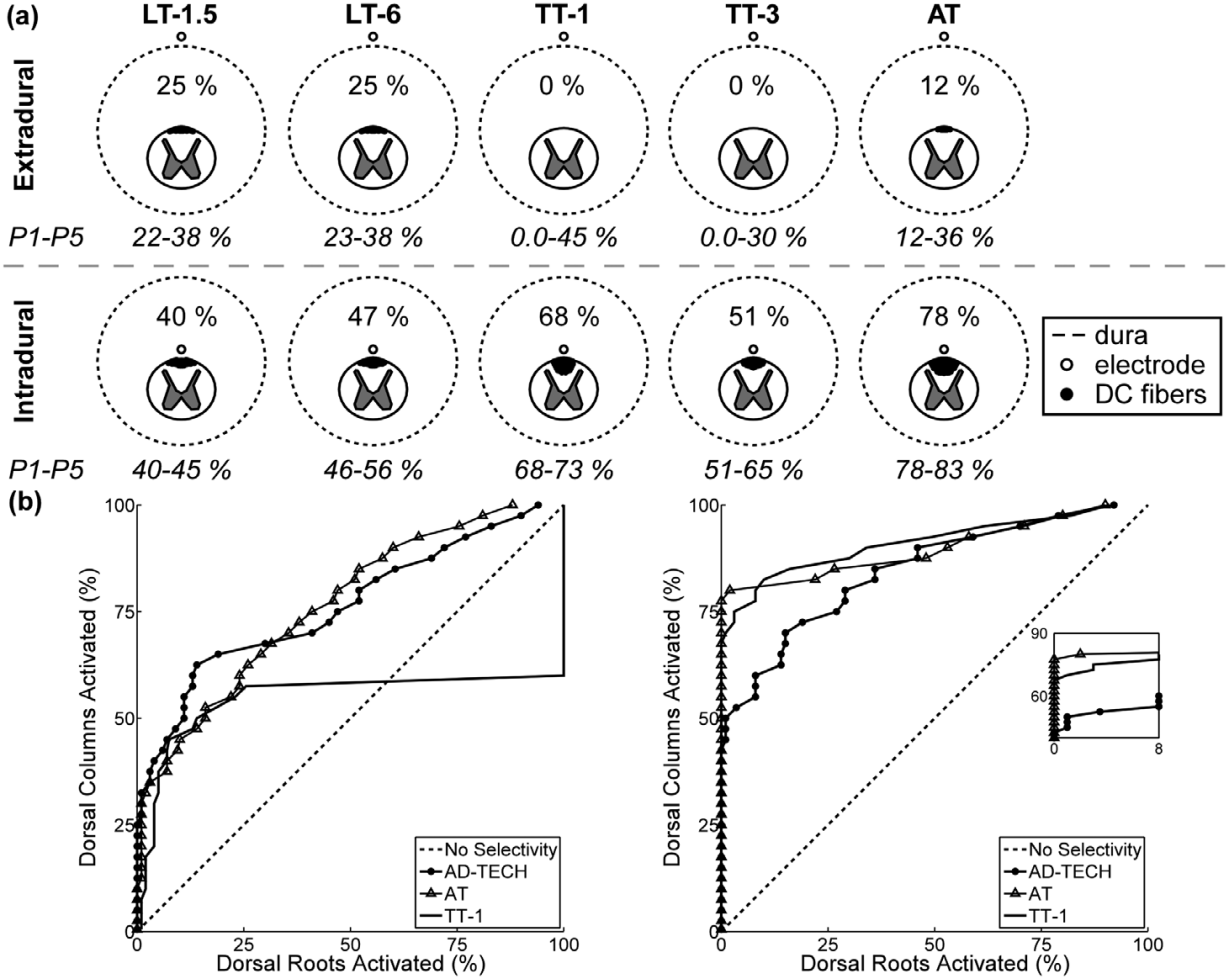
Selectivity of five tripolar electrode designs in model of Patient 2. (a) The percent of dorsal column (DC) fibers activated with no dorsal root (DR) fiber activation (*i.e.,* DC_0_) when the electrode was placed along the midline, 1 mm above (*top*) and below (*bottom*) the spinal cord. DC_0_ is shown above the spinal cord. For comparison, the range of DC_0_ across all patients is shown below each panel. (b) Proportion of DC fibers activated versus proportions of the DR fibers activated (*i.e.,* DC_X_) for three electrode designs in the extradural (*left*) and intradural (*right*) cases. The inset shows a close-up of the curves. LT = longitudinal tripolar, TT = transverse tripolar, AT = angular tripolar, and the number after the hyphen indicates the interelectrode spacing in mm.

The performance of five additional tripolar electrode designs (2 LT, 2 TT, and the AT) was tested in the SCS models of Patients 1–5. In the extradural case, LT-1.5, LT-6, TT-1, TT-3, and AT had an average (n = 5) DC_0_ of 30%, 31%, 27%, 19%, and 27%, respectively. However, the variability in DC_0_ across patients was large. For example, LT-6, which performed the best, on average, had DC_0_ that ranged from 23–38%; whereas TT-3, which performed the worst, on average, had DC_0_ that ranged from 0–30% (Fig. 10a). As a result, the distributions of the selectivities of the five designs were not significantly different from each other in the extradural case.

All tripolar electrode designs had distributions of DC_0_ in the intradural case that were significantly greater (p<0.01) than the corresponding distributions of DC_0_ in the extradural case (Fig. 10a). Further, in the intradural case, distributions of DC_0_ of the electrode designs across models of individual patients were all significantly different from each other: the AT performed better than the two TTs (p<0.01), and the two TTs performed better than the two LTs (p<0.05). Thus, selectivity in the intradural case, compared to the extradural case, was less sensitive to anatomical variations across patients.

Since the AT had the greatest neural-element selectivity in the intradural case, we also assessed its ability to activate selectively DC fibers in the lateral dermatomes. When the AT was deviated −10° from the midline, it activated DC fibers in four dermatomes, L2–S2, without activation of DC fibers in the other dermatomes; and when further deviated to −20°, the AT activated DC fibers in three dermatomes, L1–L4, without activating DC fibers in the other dermatomes. In SCS for the treatment of chronic low back pain, the target dermatomes are typically L2–L5, thus the AT had the greatest selectivity in targeting the low back dermatomes (Fig. 9b).

## Discussion

We developed a computational model of SCS and quantified the efficiency and selectivity of different electrode designs placed either extradurally or intradurally. Intradural placement dramatically increased stimulation efficiency and reduced the power required to stimulate the dorsal columns by more than 90%. Intradural placement also enabled activation of a greater proportion of dorsal column fibers before spread of activation to dorsal roots and produced more selective activation of individual dermatomes at different lateral positions. The results suggest that present electrode designs used for extradural SCS are not optimal for intradural SCS, and a novel azimuthal tripolar design increased stimulation selectivity, even beyond that achieved with an intradural paddle array in TT configurations. Increased stimulation power efficiency is expected to increase the battery lives of implantable pulse generators (IPGs), increase the recharge intervals of rechargeable IPGs, and potentially reduce IPG volume. The greater stimulation selectivity with intradural placement may improve the success rate of SCS by mitigating the sensitivity of pain relief to malpositioning of the electrode.

### The therapeutic targets predicted by the model

Models incorporating patient-specific dimensions predicted the relative order of stimulation thresholds, the greater than five-fold difference between extradural and intradural stimulation thresholds, and the sensitivity of stimulation thresholds to spinal cord position (Fig. 6). However, across these five models, there was no clear distinction of either the diameter or proportion of Aβ fiber activation that correlated best with the sensory and discomfort thresholds of the patients (Fig. 5).

Myelinated fibers in the gracilis fasciculus and cuneatus fasciculus at T3 have diameters ranging from 1–15 µm, with ∼60% between 1 and 3 µm [55]; and myelinated fibers in the gracilis fasciculus at T5 have diameters ranging from 1–7 µm, with the majority between 2 and 3 µm [53]. Prior models of extradural SCS showed that DC fibers with diameters >9.4 µm were activated between measured sensory and discomfort thresholds [21], and activation of large DC fibers with a diameter of 12 µm, which constitute <0.5% of all DC fibers [50], best matched measured sensory thresholds [21], [22]. The results of our study corroborated these prior findings in some patients (e.g., Patient 1), depending on the choice of axon model, but in the other patients (e.g., Patient 5), the results suggest that DC fibers with diameters as small as 3 µm are also activated at the sensory threshold (Figs. 5 and 6).

The discrepancy between our results and previous findings may be explained by the choice of axon model. Prior models of extradural SCS used a simplified SW model of a mammalian nerve fiber with perfectly insulating myelin [20]–[26], [40], [46]–[48], whereas we used a more detailed MRG axon model that better replicates the excitability of mammalian nerve fibers [49]. The SW model overestimated stimulation thresholds compared to the MRG model (Fig. 6), possibly explaining why prior modeling studies concluded that DC fibers with diameters >9 µm are the therapeutic targets of SCS.

The present results provide an alternate interpretation of which DC fibers are the potential therapeutic targets of SCS. Although it is possible that activation of one (or a few) 12 µm DC fibers is sufficient to evoke paresthesia [21], the results suggest that paresthesia induction requires activation of a larger proportion of DC fibers with diameters as small as 3 µm. Resolving these two possible interpretations will require more closely matching patient-specific models with the experimental conditions. For example, the presence of interstitial fluid and blood around the electrode and the distance and orientation of the stimulating array with respect to spinal cord will influence thresholds, and thereby influence the assessment of which Aβ fiber diameters are activated at sensory and discomfort thresholds. In addition, patient-to-patient variability in sensorimotor and pain networks (*i.e.,* variability in the reporter) could influence threshold sensations.

Additionally, the clinical results provide an alternate interpretation of which neural elements are responsible for discomfort. Due to the relatively low stimulation thresholds of fibers entering the DR [22], discomfort is often associated with segmental motor reflexes [56] and/or uncomfortable sensations [57] that arise from stimulation of Ia and Aβ fibers in the DR, respectively. In our study, only Patients 3 and 5 reported discomfort in T8 (Fig. 4). Although the results cannot address where discomfort occurred or what were the qualities of discomfort, they provide evidence that discomfort can be associated with activation of Aβ fibers in both target and non-target dermatomes (Fig. 4). Further, a percent increase in activation of model DC fibers was not able to explain the onset of discomfort, so other mechanisms may be required to explain discomfort. For example, discomfort may arise from stimulation of other neural elements in the DC, such as ascending nociceptive fibers or descending fibers from the brain [58], or supra-threshold depolarization of Aβ fibers, which can result in a volley of action potentials per stimulation pulse, rather than a single action potential [59].

### The design of more effective intradural SCS leads

Previous studies of extradural SCS have shown that bipolar and tripolar electrode configurations have greater selectivity than monopolar configurations [23], but the energy required for stimulation increases as the IES decreases [60]. More specifically, models of extradural SCS predict that PERC designs in longitudinal (rostrocaudal) bipolar (LB) and LT configurations have better selectivity than LAM designs in TT configurations [21], [24], although the opposite is observed in practice [18]–[20]. LAM designs are less prone to migration than PERC designs [17], and they compress the dural sac, reducing the distance between the electrodes and the spinal cord, which improves selectivity [46], [47].

Our results corroborate the predictions of previous extradural SCS studies. LT-1.5, LT-6, and the AD-TECH array in a LB configuration, on average, achieved greater DC_0_ than TT-1 and TT-3 (Figs. 8a and 10a); and energy requirements increased with decreasing IES. However, the distributions of the extradural DC_0_ of the six electrode designs across patients were not significantly different from each other, indicating that patient-to-patient variability in spinal anatomy can significantly impact the performance of the electrode. In the intradural case, the relationship between energy requirements and IES did not change, but each design had greater selectivity than the corresponding extradural case (Figs. 8a and 10a). Therefore, not only is selectivity increased with increasing proximity to the dorsal aspect of the spinal cord, but selectivity with intradural placement is less sensitive to patient anatomical variations.

**Figure 11:**
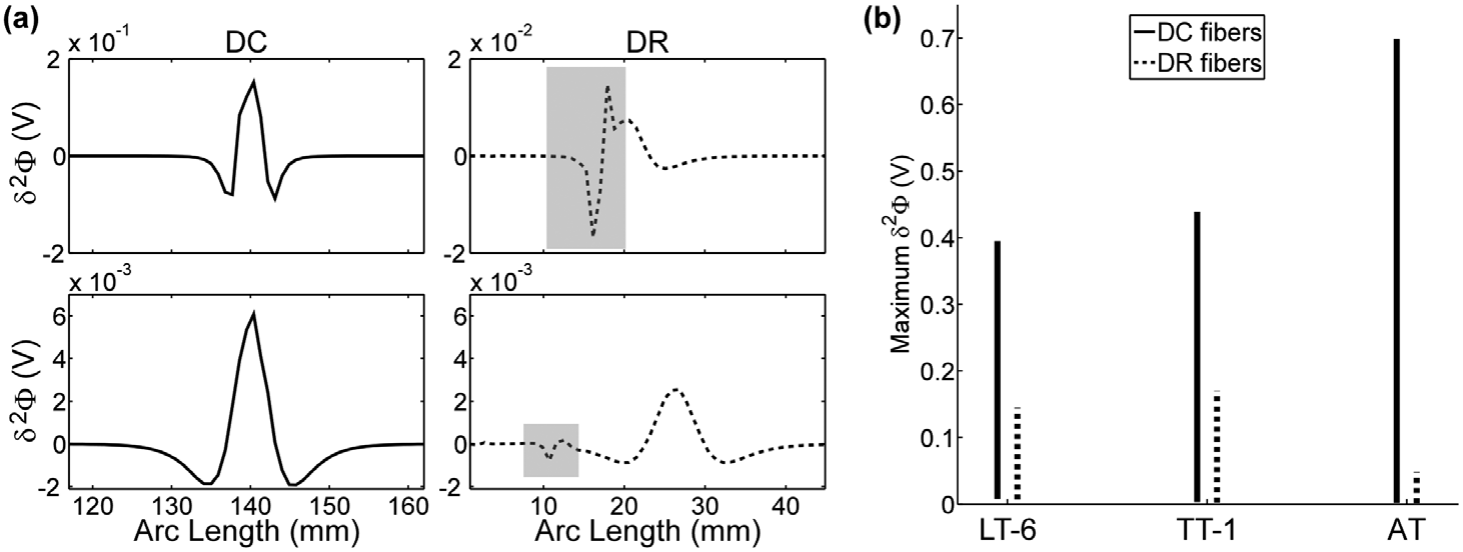
The source driving membrane polarization with three different electrode designs. (a) Examples of the centered second difference of the potentials (Δ^2^Φ) along two dorsal column (DC) fibers and two dorsal root (DR) fibers. The grey boxes indicate regions where changes in tissue conductivity caused abrupt changes in Δ^2^Φ. (b) The range of maximum Δ^2^Φ across across all modeled DC fibers and DR fibers for three electrode configurations. LT = longitudinal tripolar, TT = transverse tripolar, AT = angular tripolar, and the number after the hyphen indicates the interelectrode spacing in mm.

The AT electrode design had the greatest selectivity in the intradural case (Fig. 10). To understand why the AT performed best, we examined the centered second difference of the extracellular potentials (Δ^2^Φ) across the model fibers for each electrode design, as Δ^2^Φ is proportional to the source driving membrane polarization [61]. Across all six designs, Δ^2^Φ across DC fibers had the same stereotypical triphasic shape, including a primary positive (depolarizing) lobe flanked by two smaller negative (hyperpolarizing) lobes (Fig. 11a). All six designs also generated a triphasic Δ^2^Φ across DR fibers, except at the boundary between the CSF and the white matter, where the discontinuity in conductivity created discontinuity in Δ^2^Φ (Fig. 11a), which was expected [21]. Since no marked differences in the shape of Δ^2^Φ between the designs were observed, we conclude that the superior selectivity of the AT arose from its ability to steer current away from the spinal cord so that Δ^2^Φ decayed more rapidly with distance than it did with the LB, LT, and TT configurations. In other words, the difference in Δ^2^Φ between the DC and DR fibers was greatest with the AT electrode (Fig. 11b).

In addition to efficiency and selectivity, additional factors, such as risk and cost, should also be considered when comparing the performance of PERC and LAM designs. PERC arrays are less invasive than LAM arrays: the former are inserted using a percutaneous needle, while the latter require multi-level laminectomies for placement. Further, compared to LAM arrays, PERC arrays are less prone to fracture [17] and may be less damaging [30], as the tissue response depends on electrode size [62]. In regards to practical limitations, PERC arrays are more prone to migration and movement than LAM arrays [17], [63]. For example, rotating the AT by±10°, ±20°, and±30° about its longitudinal axis reduced the DC_0_ from 80% to 73%, 58%, and 47.5%, respectively. Reductions in pain relief that result from lead migration or movement are problematic because they may lead to greater reoperation rates to replace or reposition the lead [30]. Despite these differences, the long-term health-care costs were similar between the PERC and LAM arrays [30].

### Limitations

Two important limitations of the models used in this study require consideration. First we ignored the branching collaterals of the Aβ (DC) fibers. As the Aβ fibers ascend the spinal cord to the gracile and cuneate nuclei in the brainstem, they project smaller diameter collaterals to neurons within the grey matter of the spinal cord [64]. This branching can reduce stimulation thresholds by up to 50% in SW models of DC fibers [48], and the stimulation thresholds reported in the present study may therefore be overestimated.

The second limitation was ignoring the presence and properties of the electrode-tissue interface (ETI). The filtering effects of the ETI have not been studied in SCS, but they have been studied in electrical stimulation of the brain [65]–[67]. The ETI, which is typically modeled as the parallel combination of a distributed resistor and a distributed capacitor, has a time constant on the order milliseconds. Because typical pulse widths for SCS range from 175–600 µs, the ETI is expected to charge by an appreciable amount during the stimulus pulse, increasing the dynamic load on the stimulator. The rate at which the dynamic load increases depends on the electrical properties of the ETI and tissue, which depend on electrode geometry. Thus, representation of the ETI is recommended for future studies comparing the efficiency of SCS electrode designs.

In addition to model limitations, this study was also limited by experimental variability in the clinical study. Sources of uncertainty included variability in electrode placement and position and variability in the experimental conditions. For example, in Patient 1, intradural stimulation thresholds were assessed before extradural stimulation thresholds, while the order was reversed in Patients 2–5. Because the dura of Patient 1 was punctured before extradural assessment, leakage of CSF might explain why the extradural clinical thresholds of Patient 1 were smaller than those of Patients 2–5 (Fig. 6). Experimental variability can be reduced in future studies by standardizing all aspects of the clinical procedure and using intraoperative fluoroscopy images to determine more accurately the position of the electrode during stimulation.

## Conclusion

The present study used a computational model of SCS to evaluate quantitatively the performance of intradural SCS for treating chronic pain. Intradural electrode placement markedly reduced energy consumption and improved selectivity of activation of DC fibers in both medial and lateral dermatomes without co-activation of DR fibers. Further, the results suggest that DC fibers with diameters as small as 3 µm are activated within the therapeutic range of SCS parameters, challenging the notion that only DC fibers with diameters >9 µm are activated in SCS. More anatomical studies are needed to characterize better the distribution of fiber diameters within the DC so that subsequent modeling studies can more accurately quantify the population of Aβ fibers that correspond to evoking paresthesia and discomfort; and long-term clinical studies are needed to test the predications of our model, understand better the percentage and diameter of neural elements that correspond to comfortable and uncomfortable sensations, and assess the potential therapeutic benefits of intradural SCS.
