# Correction: Evaluation of Intradural Stimulation Efficiency and Selectivity in a Computational Model of Spinal Cord Stimulation

**DOI:** 10.1371/journal.pone.0123485

**Published:** 2015-04-07

**Authors:** The PLOS ONE Staff

The Data Availability statement for this paper is incorrect. The correct statement is: The authors confirm that all data underlying the findings are fully available without restriction. All relevant data are deposited in the Harvard Dataverse Network, with the DOI:10.7910/DVN/29236.

## References

[1] Howell B, Lad SP, Grill WM (2014) Evaluation of Intradural Stimulation Efficiency and Selectivity in a Computational Model of Spinal Cord Stimulation. PLoS ONE 9(12): e114938 doi: 10.1371/journal.pone.0114938 25536035 10.1371/journal.pone.0114938 PMC4275184

